# Fermi-arc supercurrent oscillations in Dirac semimetal Josephson junctions

**DOI:** 10.1038/s41467-020-15010-8

**Published:** 2020-03-02

**Authors:** Cai-Zhen Li, An-Qi Wang, Chuan Li, Wen-Zhuang Zheng, Alexander Brinkman, Da-Peng Yu, Zhi-Min Liao

**Affiliations:** 1 State Key Laboratory for Mesoscopic Physics and Frontiers Science Center for Nano-optoelectronics, School of Physics, Peking University, Beijing, 100871 China; 2 Shenzhen Institute for Quantum Science and Engineering and Department of Physics, Southern University of Science and Technology, Shenzhen, 518055 China; 3 Academy for Advanced Interdisciplinary Studies, Peking University, Beijing, 100871 China; 4 MESA+ Institute for Nanotechnology, University of Twente, 7500 AE Enschede, The Netherlands; 5 Beijing Key Laboratory of Quantum Devices, Peking University, Beijing, 100871 China; 6 Collaborative Innovation Center of Quantum Matter, Peking University, Beijing, 100871 China

**Keywords:** Electronic properties and materials, Superconducting properties and materials, Topological insulators

## Abstract

One prominent hallmark of topological semimetals is the existence of unusual topological surface states known as Fermi arcs. Nevertheless, the Fermi-arc superconductivity remains elusive. Here, we report the critical current oscillations from surface Fermi arcs in Nb-Dirac semimetal Cd_3_As_2_-Nb Josephson junctions. The supercurrent from bulk states are suppressed under an in-plane magnetic field ~0.1 T, while the supercurrent from the topological surface states survives up to 0.5 T. Contrary to the minimum normal-state conductance, the Fermi-arc carried supercurrent shows a maximum critical value near the Dirac point, which is consistent with the fact that the Fermi arcs have maximum density of state at the Dirac point. Moreover, the critical current exhibits periodic oscillations with a parallel magnetic field, which is well understood by considering the in-plane orbital effect from the surface states. Our results suggest the Dirac semimetal combined with superconductivity should be promising for topological quantum devices.

## Abstract (web summary)

Superconductivity through the topological surface states of a Dirac semimetal remains elusive. Here, the authors fabricate a Nb-Cd_3_As_2_-Nb Josephson junction and observe supercurrent oscillations from surface Fermi arcs of Cd_3_As_2_

## Introduction

Materials with topological surface states have become one of the most intensive fields of condensed matter research in past years^1–3^. Among the various topological materials, the topological semimetal has sparked substantial interest due to its gapless Weyl/Dirac cones and unique surface Fermi arcs^3–5^. With nontrivial Fermi-arc surface states^5–9^, the Dirac semimetal Cd_3_As_2_ has demonstrated exotic quantum transport properties of these surface states, such as π Aharonov–Bohm effect^9,10^, Fermi-arc-mediated Weyl orbital transport^11,12^, and quantum Hall effect from topologically protected Fermi arcs^13–17^. Besides the transport research in its normal phase, efforts have recently been made to couple the Fermi-arc surface states to a superconductor with the expectation of Majorana fermions^18–21^. Such proximitized superconductivity has been observed in Cd_3_As_2_, including surface carried Josephson supercurrent^22^, π and 4π Josephson effects^23,24^. For the potential control of Majorana fermions and real-life application of topological quantum computation, it is of great necessity to establish a good manipulation over the superconducting Fermi-arc states.

Here, we report the magnetic field and gate modulation of the Fermi-arc superconductivity in Nb-Cd_3_As_2_-Nb Josephson junctions. Without magnetic field, the supercurrent is carried by both bulk and surface states. With increasing an in-plane magnetic field, the bulk-carried supercurrent is strongly suppressed and the Fermi-arc surface states become manifest. In the surface state dominant regime, the critical supercurrent shows a maximum value near the Dirac point, consistent with the fact that the Fermi arcs have the maximum density of states at the Dirac point. The maximum critical supercurrent at Dirac point in 3D Dirac semimetal is different from the case of 2D Dirac states in topological insulators and graphene. Furthermore, the Fermi-arc supercurrent shows periodic oscillations with in-plane parallel magnetic field, which is attributed to the in-plane field orbital interference of the surface Fermi arcs. Such magnetic field and gate modulation of superconducting Fermi arcs open up a new avenue for the manipulation of Majorana fermions, which might be significant to the topological quantum computation.

## Results

### Andreev reflections in the Dirac semimetal Josephson junction

**Fig. 1:**
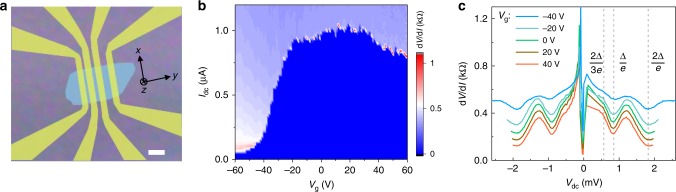
Josephson effect in a Nb-Cd_3_As_2_-Nb junction. **a** Optical image of the Nb-Cd_3_As_2_ nanoplate-Nb Josephson junctions. Scale bar, 2 μm. **b** The color-scale differential resistance d*V*/d*I* as a function of gate voltage *V*_g_ and d.c bias current *I*_dc_. **c** The d*V*/d*I* versus source-drain voltage *V*_dc_ across the junction, showing the multiple Andreev reflections.

The Josephson junctions consist of Cd_3_As_2_ nanoplates and superconducting Nb electrodes (Fig. 1a). The Cd_3_As_2_ nanoplates are of high crystalline quality with (112) oriented surfaces (Supplementary Fig. 1). Individual Cd_3_As_2_ nanoplates were transferred into a silicon substrate with a SiO_2_ layer (285 nm), which serves as the back gate. The separation length *L* between the two Nb electrodes is about 300 nm for the measured junction presented in the main text. The average width *W* of the nanoplate is 5 μm. The flake thickness *t* is about 80 nm. Electrical transport measurements were performed in a dilution refrigerator with a base temperature of 12 mK.

Figure 1b shows the differential resistance (d*V*/d*I*) as a function of current bias *I*_dc_ and gate voltage (*V*_g_). A gate tunable nondissipative supercurrent is observed. As tuning *V*_g_ from 60 to −60 V, the critical current *I*_c_ first increases and reaches a maximum value of 1 μA at around *V*_g_ = 20 V, and then decreases rapidly to about 50 nA when *V*_g_ < −50 V. The strong suppression of *I*_c_ at negative *V*_g_ is due to the low hole mobility of bulk states in Cd_3_As_2_ (ref. ^25^). The behavior of *I*_c_ peak at *V*_g_ = 20 V is resulted from the coexistence of bulk and surface states as demonstrated later. In Fig. 1c, we show the d*V*/d*I* as a function of the bias voltage (*V*_dc_) between two superconducting electrodes at different gate voltages. A series of dips in d*V*/d*I* spectra at *V*_n_ = 2Δ/*n*e (*n* = 1, 2…) are attributed to the multiple Andreev reflections. The induced superconducting gap is estimated to be 0.9 meV, which is smaller than the gap value of the Nb layers (1.4 meV).

### Supercurrent oscillations under in-plane magnetic field

**Fig. 2:**
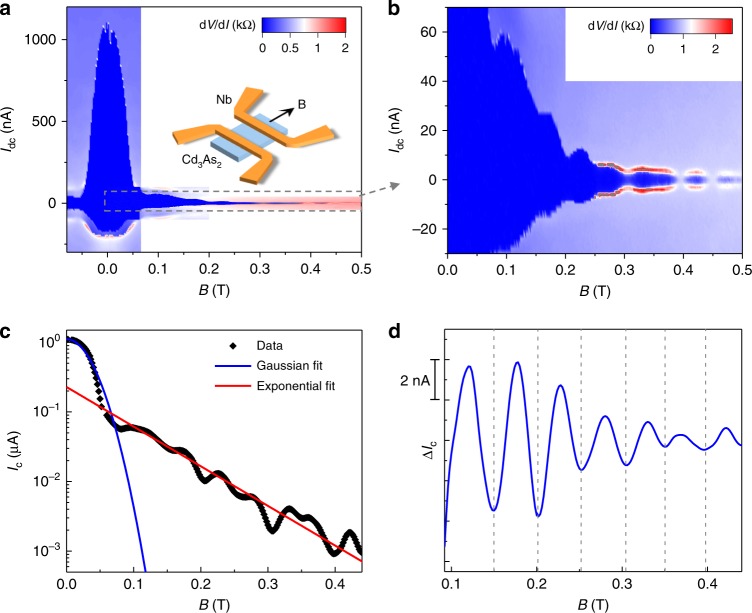
The supercurrent oscillations under parallel magnetic field at *V*_g_ = 0 V. **a** The d*V*/d*I* as a function of magnetic field **B** and *I*_dc_. The *I*_dc_ is swept from negative to positive. The applied excitation current *I*_ac_ = 0.5 nA. Inset: Schematic of the magnetic field direction on the junction. **b** The enlarged d*V*/d*I* map of the gray dotted box in **a**. Periodic supercurrent oscillations with multiple nodes are observed. **c** The magnetic field dependence of *I*_c_ with a semilog coordinate. The Gaussian fitting (blue curve) well models the decay trend of *I*_c_ at low **B**, and an exponential decay (red line) fits better the data for **B** > 0.1 T. **d** The extracted Δ*I*_c_ by subtracting a smooth background as a function of **B**. A period of Δ**B** = 0.05 T is obtained from the oscillations.

When an in-plane magnetic field **B** is applied parallel to the current direction, the critical current *I*_c_ first shows a rapid decay, and then oscillates periodically as a function of **B**. Figure 2a shows a typical spectrum of d*V*/d*I* as a function of **B** and *I*_dc_. The *I*_c_ decreases from 1.1 μA to ~65 nA as increasing **B** from 0 to 70 mT. The *I*_c_ then exhibits an oscillating behavior until 0.5 T (Fig. 2b).

The critical current of a diffusive thin film is expected to decrease monotonically in a parallel magnetic field with $I_c\left( {\mathbf{B}} \right) \approx I_{c}(0)e^{ - {\mathbf{B}}^2/2\sigma ^2}$, like a Gaussian function^26^, where *σ* is the decay coefficient. A Gaussian fit can well describe the *I*_c_ trends under low field, but obviously fails in the case of high field, where *I*_c_ is suppressed with a much lower rate (Fig. 2c). A kink behavior is clearly observed near 0.1 T, which separates the two different drop rates of *I*_c_ under low and high magnetic fields. This implies that two channels (bulk and surface) coexist and response differently to magnetic field. Under zero magnetic field, there is an unavoidable coexistence of bulk and surface states due to the highly conductive bulk and large surface-to-volume ratio in nanostructured Cd_3_As_2_. When applying a magnetic field, the supercurrent from the bulk states is strongly suppressed, while the supercurrent from surface states can still survive up to 0.5 T benefiting from the topological nature and protection from backscattering. Thus the surface states are responsible for the supercurrent under high magnetic field that decays with a much lower rate. After subtracting the decay background under high magnetic field, the plot of Δ*I*_c_ with **B** demonstrates periodic oscillations with a period of Δ**B** ~ 0.05 T, as shown in Fig. 2d.

### Gate tuned critical supercurrent carried by Fermi arcs

**Fig. 3:**
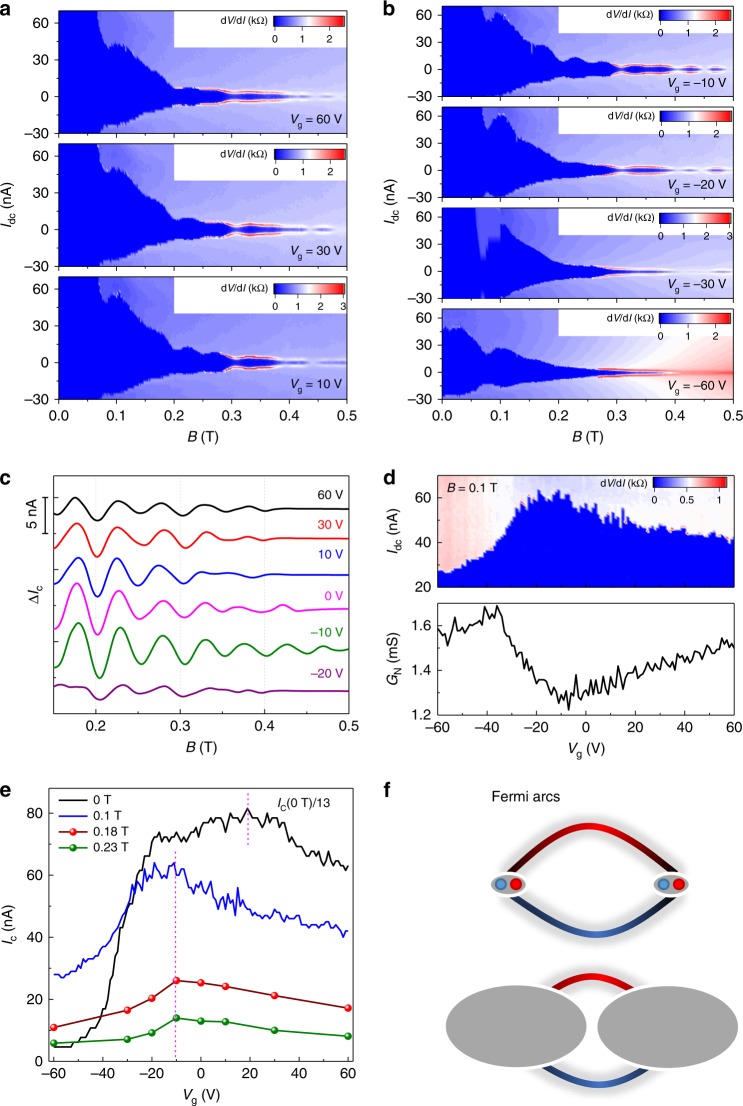
Gate dependence of supercurrent oscillations. **a, b** Color-scale plot of d*V*/d*I* as a function of **B** and *I*_dc_ at different *V*_g_ as denoted. **c** The extracted Δ*I*_c_ versus **B** at different *V*_g_. The curves have been shifted for clarity. **d** The comparison between critical current *I*_c_ and normal-state conductance *G*_N_ as a function of *V*_g_, measured at **B** = 0.1 T. **e** *I*_c_(*V*_g_) evolutions under different magnetic fields. The *I*_c_(0T) divided by 13 is shown in the figure. The *I*_c_(0.18T) and *I*_c_(0.23T) are extracted from the *I*_*c*_(*B*) peaks of the first and second oscillation lobes in **c**, respectively. **f** The Fermi arcs for the Fermi level (up panel) close to and (bottom panel) away from Dirac point. The bulk Dirac points are projected on (112) crystal plane of the Cd_3_As_2_ nanoplate.

The *I*_c_ oscillations are further investigated by tuning the Fermi level of the Cd_3_As_2_ nanoplate. Figure 3a, b show a series of d*V*/d*I* as a function of **B** and *I*_dc_ at different values of *V*_g_. As varying *V*_g_, the oscillation period Δ**B** remains unchanged with discernible oscillating nodes, as marked by the uniformly spaced dashed lines in Fig. 3c. The constant period as a function of the gate voltage indicates that the critical current oscillations are insensitive to the carrier density. Figure 3d shows the comparison between *I*_c_ and normal-state conductance *G*_N_ (measured at *I*_dc_ = 100 nA) under **B** = 0.1 T. Near the Dirac point, the *G*_N_ reaches a minimum, while *I*_c_ unexpectedly acquires a maximum value, indicating that the dominant conduction channels for the superconducting and normal states are different.

The coexistence of bulk and surface states is reflected by the *I*_c_ evolution with magnetic field (Fig. 3e). Under zero field, the *I*_c_ shows a rapid increase as tuning the Fermi level from the hole conduction region to the Dirac point, while increases slightly with further increasing gate voltage to 20 V, and then shows a downward trend for *V*_g_ > 20 V. Considering the screening of gate electric field at large *V*_g_, the inhomogeneous carrier distribution may break the Andreev pairs and reduce the *I*_c_. Since the bulk pairing can be greatly suppressed by magnetic field, the *I*_c_ is significantly reduced under 0.1 T, and a *I*_c_ peak appears near the Dirac point. Further increasing magnetic field to 0.18 and 0.23 T, the position of the *I*_c_ peak keeps unchanged at around *V*_g_ = −10 V, indicating a fully surface state dominant regime. In Cd_3_As_2_, the surface states are in the form of Fermi arcs, which connect the projection of two bulk Dirac points on the surface. As tuning the bulk Fermi level close to the Dirac point, the proportion of Fermi arc would acquire a maximum value (Fig. 3f). Thus, in a surface dominant regime, the Fermi arc carried supercurrent would acquire a maximum value near the Dirac point. Moreover, at the Dirac point, the density of state of bulk is minimum, and thus the less scattering from the bulk state also facilitates the Fermi-arc supercurrent^22^. The Fermi-arc supercurrent survives at higher magnetic field, which is attributed to the topological protection and long phase coherence length of the Andreev pair states.

**Fig. 4:**
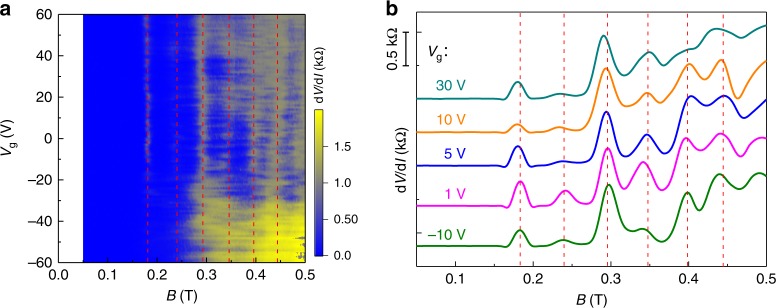
The evolution of differential resistance with magnetic field B and *V*_g_. **a** The d*V*/d*I* as a function of **B** and *V*_g_ with *I*_ac_ = 1 nA and without applying *I*_dc_. The vertical dashed lines are eyes guided. **b** The cut lines of d*V*/d*I* as a function of **B** extracted from **a** at *V*_g_ = 30, 10, 5, 1, and −10 V, respectively. The curves have been shifted for clarity. The d*V*/d*I* peaks emerge periodically with a period of Δ**B** = 0.055 T.

The superconducting state transition and its gate dependence are further studied by the measurement of d*V*/d*I* as a function of **B** and *V*_g_ with *I*_ac_ = 1 nA and without applying *I*_dc_. Figure 4 shows that the superconducting state also exhibits an oscillating pattern with increasing **B**. In the whole range of magnetic field upto 0.5 T, six distinct superconducting regions are clearly separated, as marked in Fig. 4a by the red dashed lines. To highlight the periodically reentrant behavior of the superconducting state, the d*V*/d*I* as a function of **B** at different *V*_g_ is plotted in Fig. 4b. The junction transforms from the superconducting state to normal state at around **B** ~ 0.19 T. With further increasing **B**, the system then reenters into the superconducting state. The d*V*/d*I* peaks are nearly periodic in **B** with a period of around 0.055 T, which is consistent with the *I*_c_ oscillation period (0.05 T). The superconducting state always exists until 0.19 T (Fig. 4a), which is mainly due to the fact that the bulk states can carry supercurrent in low field and is consistent with the nonzero critical current at the *I*_c_ oscillation nodes in low field (Fig. 2b).

## Discussion

From the above results, we can conclude that the supercurrent is carried mainly by the Fermi-arc surface states of the nanoplate under high magnetic field. Next we would like to discuss the possible mechanisms of the supercurrent oscillations with magnetic field. Recent studies show that, in certain materials, the mechanism of finite momentum Cooper pairing can give rise to extra superconducting coherence and spatially oscillating parameter when subjected to in-plane magnetic exchange fields^27–29^. Critical current oscillations in superconductor–ferromagnet–superconductor junctions have provided evidences for both nonzero pairing momentum and 0–π transition^30–32^. More recently, quantum oscillations arising from in-plane Zeeman field induced finite momentum pairing have been demonstrated in Josephson systems of a Bi nanowire^33^, topological insulators^34–36^, and a Bi_0.97_Sb_0.03_ topological semimetal^37,38^.

In Dirac semimetal Cd_3_As_2_, each Dirac cone splits into two Weyl cones along the direction of the magnetic field^39^ (Supplementary Fig. 2). Because of the shift, the Andreev pair states will gain a finite center of mass momentum ${\mathrm{\Delta }}k = \frac{{g\mu _{\mathrm{B}}{\mathbf{B}}}}{{\hbar v_{\mathrm{f}}}}$, where *g* is the Landé factor, *μ*_B_ is the Bohr magneton, *ħ* is the reduced Planck constant, and *v*_f_ is the Fermi velocity. The finite momentum results in a dephasing of the superconducting pairing potential and eventually modulates the critical current periodically in magnetic field^27,28^. The oscillation period in magnetic field satisfies the relation Δ*k* × *L* = π. Using an averaged *g* = 30 as reported in literatures^40^ and Fermi velocity *v*_f_ = 5 × 10^5^ m*s*^−1^ we obtain the expected period Δ**B** ~ 1.98 T which is around 40 times larger than the measured period 0.05 T. This means the Zeeman effect is not likely to be the dominant cause of the supercurrent oscillations. Spin–orbit coupling (SOC) can also give rise to an anomalous momentum shift and thereby oscillatory patterns^36,41^. However, the SOC-related momentum shift requires the field in-plane perpendicular to the current, which does not apply to our case.

If there is a small perpendicular component of the applied magnetic field due to misalignment, the conventional Fraunhofer diffraction pattern may come into effect^42^. The junctions on the same nanoplate should have similar Fraunhofer patterns and the oscillation period should be proportional to the 1/*L*. However, Junction B (*L* = 500 nm) in the same nanoplate shows a longer oscillation period than that of Junction A (*L* = 300 nm) (Supplementary Fig. 3). Therefore, the effect of Fraunhofer diffraction pattern can be simply ruled out. To further exclude the effect of possible perpendicular field components, we have also studied the critical current oscillations under an in-plane magnetic field perpendicular to the current direction (Supplementary Fig. 4). With the increase of channel length *L*, the location of the first node shifts to lower magnetic field. Such a length dependence of critical current oscillations is consistent with the Fraunhofer diffraction pattern, while is sharply contrasted to that for a parallel magnetic field. Therefore, the contamination of perpendicular field components can be safely ruled out. In this way, the possible interference effects related to in-plane perpendicular fields, including SOC-induced momentum shift and SQUID-like interference, can also be easily excluded as the cause of supercurrent oscillations.

**Fig. 5:**
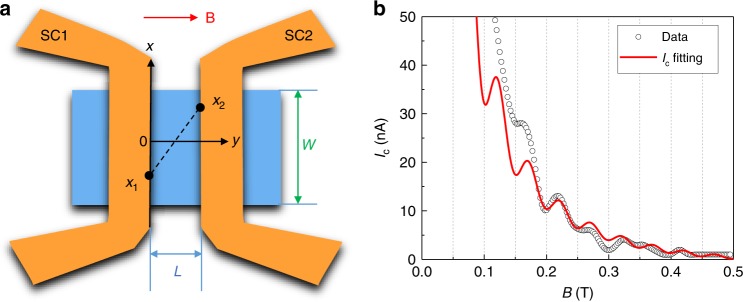
Modeling Josephson interference in regime of in-plane field orbital effect. **a** Within two superconducting (SC) leads, pairing electrons traverse from position *x*_1_ of SC1 to position *x*_2_ of SC2, accumulating phase *ϕ*_2_(*x*_2_) − *ϕ*_1_(*x*_1_). **b** The fit of *I*_c_ for **B** > 70 mT using the model of surface in-plane field orbital effect. The experimental data are from the *I*_c_(**B**) at *V*_g_ = −10 V.

It has been reported that the in-plane orbital interference can also induce the critical current oscillations^34–36^. As illustrated in Fig. 5a, we can model the phase difference *ϕ*_1_(*x*_1_) − *ϕ*_2_(*x*_2_) of the superconducting pairs, arising from the in-plane field orbital effect^35^:

1 $$\phi _1\left( {x_1} \right) - \phi _2\left( {x_2} \right) = \frac{{\pi {\mathbf{B}}\left( {x_1 - x_2} \right)t}}{{{\mathrm{\Phi }}_0}},$$

where *t* is the thickness of nanoplate, and *ϕ*_1_(*x*_1_) and *ϕ*_2_(*x*_2_) are the phases of the order parameters of superconductors 1 and 2 at the position *x*_1_ and *x*_2_, respectively, along the width of the junction. For a bulk pairing state, the trajectory traverses the whole bulk and the total integration of the accumulated phase gives a negligible net phase shift, which only results in a *I*_c_ decay without oscillations (Supplementary Fig. 5). For a surface pairing state, on the other hand, the trajectory will come along the circumferential direction of the flake. The surface related supercurrent can be expressed as^35^:

2 $$I^{{\mathrm{surface}}}\left( {{\mathrm{\Delta }}\phi ,{\mathbf{B}}} \right) = \int_{-\frac{W}{2}}^{\frac{W}{2}} \int _{-\frac{W}{2}}^{\frac{W}{2}} dx_1dx_2\frac{1}{{{\mathbf{r}}^\varepsilon }}\sin \left( {{\mathrm{\Delta }}\phi + \phi _1\left( {x_1} \right) - \phi _2\left( {x_2} \right)} \right),$$

where *W* is the junction width, ${\mathbf{{\it{r}}}} = \sqrt {L^2 + \left( {x_1 - x_2} \right)^2}$ is the distance between two point (*x*_1_, *x*_2_), and *ε* denotes the phase coherent strength along the *x* direction (Supplementary Fig. 6). Considering the magnetic screening effect from superconducting electrodes (Supplementary Fig. 7), the devices experienced magnetic field is smaller than the applied field, which is denoted by *α***B** *(α* < 1). The critical current is defined as the maxima in one period of 2π phase, *I*_c_(**B**) = max[I(Δ*ϕ*, **B**)]. As shown in Fig. 5b, the oscillating *I*_c_ under high magnetic fields can be well fitted by the model of surface in-plane field orbital effect and the fitting results give the parameters *ε* = 0.22 and *α* = 0.2.

From the modeling and fitting results, we can conclude that the supercurrent is carried mainly by the surface states of the nanoplate under high magnetic field. The periodic critical current oscillations can be understood by considering the in-plane orbital effect. This work provides a flexible gate and magnetic field manipulations of Fermi-arc superconductivity. Compared with the finite momentum pairing observed in topological insulator^35^, we would like to clarify the differences between our work and that in Bi_2_Se_3_. First, Bi_2_Se_3_ and Cd_3_As_2_ belong to different topological phases, that is, Bi_2_Se_3_ is a strong topological insulator protected by the time-reversal symmetry, while Cd_3_As_2_ is a 3D Dirac semimetal with an extra C_4_ rotational symmetry. In addition, the surface nature of the two systems is topologically different. The surface states of a Dirac semimetal are in the form of Fermi arcs, in stark contrast with the Fermi surface in topological insulator surface. As the Fermi arcs connect the surface projection points of the Weyl nodes, the density states of Fermi arcs can be tuned by tuning the bulk Fermi level. A maximum critical supercurrent is observed near Dirac point, which is totally different from the 2D Dirac systems of graphene and topological insulator surface. Especially, the Dirac semimetals transform into Weyl semimetals as applying magnetic field to break the time-reversal symmetry. The Fermi arcs start to deform and chirality-related polarization arises (Supplementary Fig. 8), providing a good platform for the investigation of superconductivity of chiral polarized states.

## Methods

### Sample synthesis

High quality Cd_3_As_2_ nanoplates were synthesized by chemical vapor deposition method^43^. Cd_3_As_2_ powders with high purity (>99.99%) were placed in the center of horizontal quartz tube. Silicon wafers with 5 nm gold thin film were placed downstream as substrates to collect the products. The quartz tube was first flushed three times with Argon gas to get out of oxygen, then gradually heated from room temperature to 700 °C within 20 min, and kept for 10 min at 700 °C along with an Argon gas flow of 20 s.c.c.m. The system was then cooled down naturally. The products of Cd_3_As_2_ nanoplates were collected on the silicon wafer substrates.

### Device fabrication

Individual Cd_3_As_2_ nanoplate was transferred into silicon substrates with an oxide layer (SiO_2_, 285 nm). The nanoplate thickness *t* is about 80 nm. After a series process of standard e-beam lithography and Ar^+^ plasma etching, Nb/Pd electrodes (100 nm/2 nm) were deposited in situ by sputtering.

### Transport measurement

Transport measurements were performed in a dilution refrigerator (Oxford Instruments Triton 200) with a base temperature ~12 mK. With the use of standard lock-in technique (SR830) in the pseudo-four-probe current–voltage geometry, the electrical signals were acquired. The differential resistance (d*V*/d*I*) was measured by applying a small a.c bias current *I*_ac_ (typically in the range of 0.5–5 nA for different sweeping range) and concurrently measuring the a.c voltage. For the measurement of critical currents, a d.c bias signal *I*_dc_ was superimposed on the *I*_ac_.

## Supplementary information

Supplementary Information

## Data Availability

The data that support the findings of this study are available from the corresponding author upon reasonable request.
