# Supplementary material for: Fermi-arc supercurrent oscillations in Dirac semimetal Josephson junctions
Source: Nat Commun. 2020 Mar 2;11:1150. doi: 10.1038/s41467-020-15010-8 (PMC7051961; doi:10.1038/s41467-020-15010-8)
Supplement: Supplementary file 1 — Supplementary Information [file 41467_2020_15010_MOESM1_ESM.pdf]

Supplementary Information for

Fermi-arc supercurrent oscillations in Dirac semimetal Josephson junctions

Cai-Zhen Li,[1,2#] An-Qi Wang,[3#] Chuan Li,[4*] Wen-Zhuang Zheng,[1] Alexander Brinkman,[4] Da-Peng Yu,[2] Zhi-Min Liao[1,5,6*]

[1]*State Key Laboratory for Mesoscopic Physics and Frontiers Science Center for Nano-optoelectronics, School of Physics, Peking University, Beijing 100871, China.*

[2]*Shenzhen Institute for Quantum Science and Engineering and Department of Physics, Southern University of Science and Technology, Shenzhen 518055, China.*

[3]*Academy for Advanced Interdisciplinary Studies, Peking University, Beijing 100871, China.*

[4]*MESA+ Institute for Nanotechnology, University of Twente, 7500 AE Enschede, The Netherlands.*

[5]*Beijing Key Laboratory of Quantum Devices, Peking University, Beijing 100871, China.*

[6]*Collaborative Innovation Center of Quantum Matter, Peking University, Beijing 100871, China.*

#These authors contributed equally: Cai-Zhen Li, An-Qi Wang.

* Email: liaozm@pku.edu.cn ; chuan.li@utwente.nl

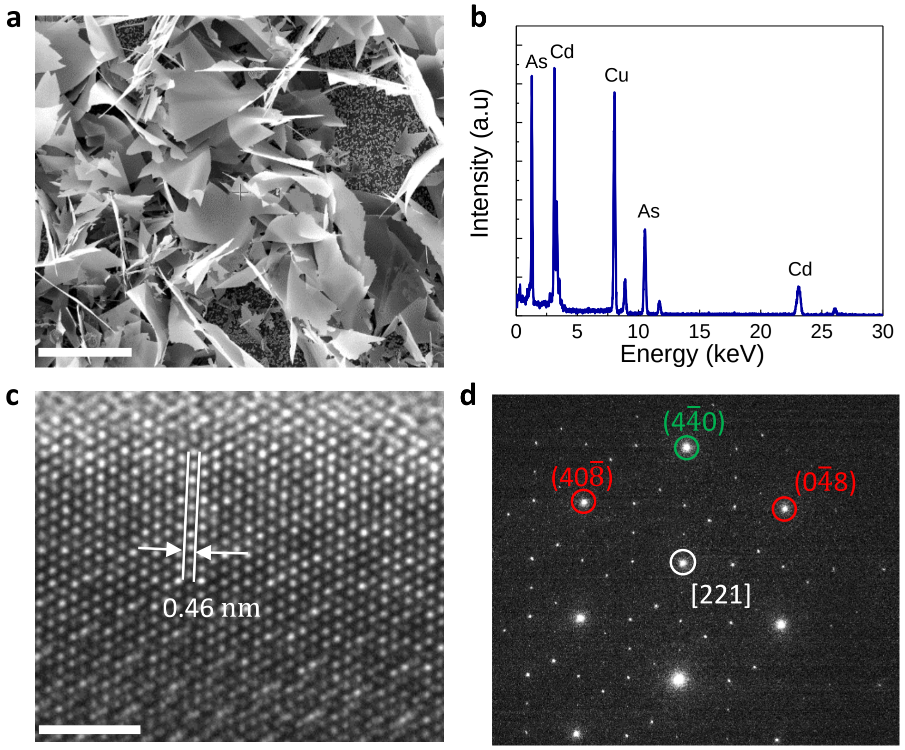

Supplementary Figure 1 | Characterization of the synthesized Cd_3As_2 nanoplates. (**a**) SEM image of the nanoplates. Scale bar, 40 μm. (**b**) The energy-dispersive X-ray spectroscopy (EDS) spectrum of the nanoplate. The quasi-quantitative analysis shows that the atomic ratio of Cd to As is consistent with the chemical composition of Cd_3As_2. (**c**) The high resolution TEM image of a typical nanoplate. The 0.46 nm interplanar spacing indicates the $(1\bar{1}0)$ edge direction of the nanoplate. Scale bar, 2 nm. (**d**) The selected electron diffraction (SAED) pattern clearly shows the hexagonal symmetry of the (112) surface plane with [221] zone axis.

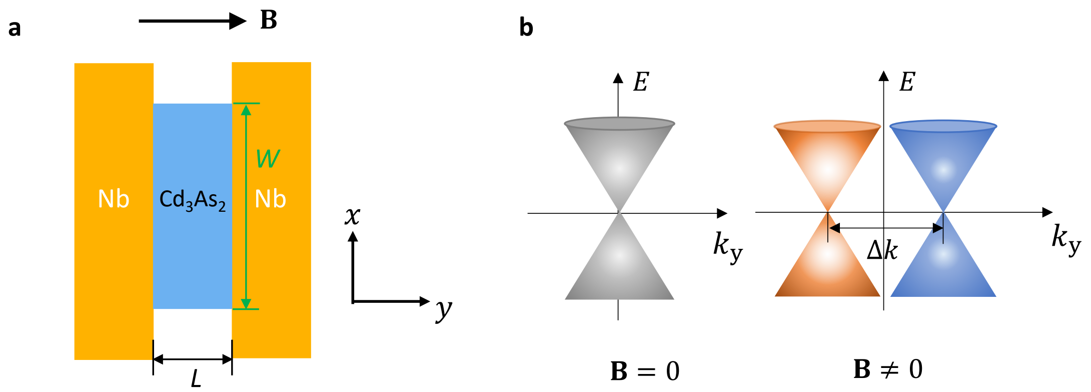

Supplementary Figure 2 | Zeeman effect on the momentum shift. (**a**) Schematic of the device in real space. (**b**) Splitting of Dirac point into two Weyl points when applying a magnetic field **B**.

Due to the linear dispersion relation in Dirac semimetals, the Zeeman effect shifts the band structure linearly in momentum space. When an in-plane magnetic field **B** is applied, each Dirac point splits into two Weyl points with opposite chirality along the direction of **B**, as shown in Supplementary Fig. 2b. The momentum shift Δk between the Weyl points is proportional to the Zeeman energy, given by $\Delta k = \frac{g\mu_{\mathrm{B}}\mathbf{B}}{\hbar v_{\mathrm{f}}}$, in which g is the Landé factor, μ_{B} is the Bohr magneton, $\hbar$ is the reduced Planck constant, and v_{f} is the Fermi velocity.

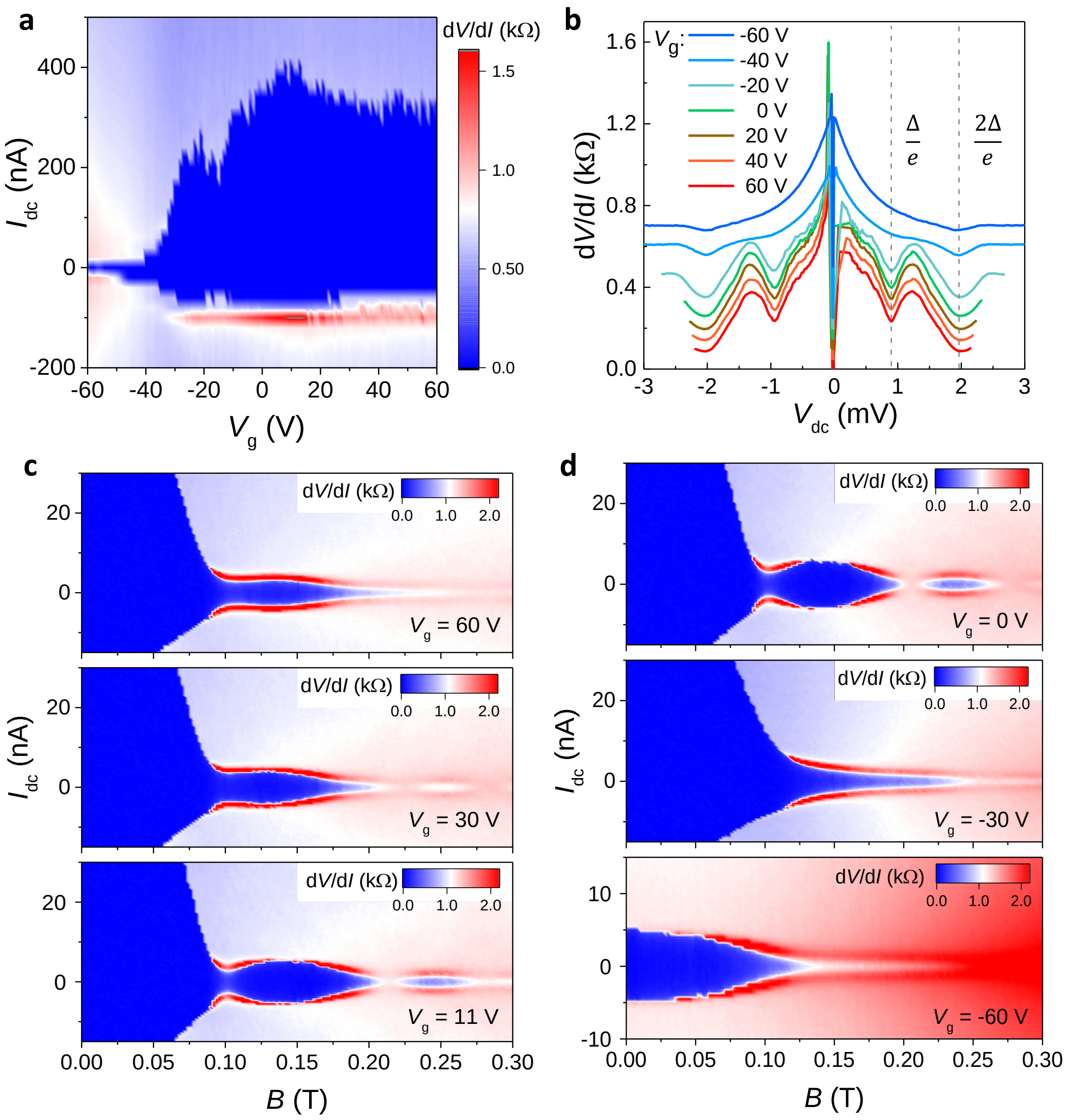

Supplementary Figure 3 | Josephson supercurrent of Junction B with L = 500 nm. (**a**) Differential resistance dV/dI as a function of V_g and I_{dc} measured at a base temperature of 12 mK. Under negative gate voltages, the critical current is greatly reduced. (**b**) The dV/dI versus source-drain voltage V_{dc} across the junction, showing the multiple Andreev reflections below the induced superconducting gap, 2Δ = 1.8 meV. (**c-d**) Critical current oscillations at different gate voltages. The I_c lobes are much more evident at V_g = 11 and 0 V. The oscillating period in Juntion B is about 0.1 T, which is larger than the 0.05 T period of the Junction A with L = 300 nm presented in the main text.

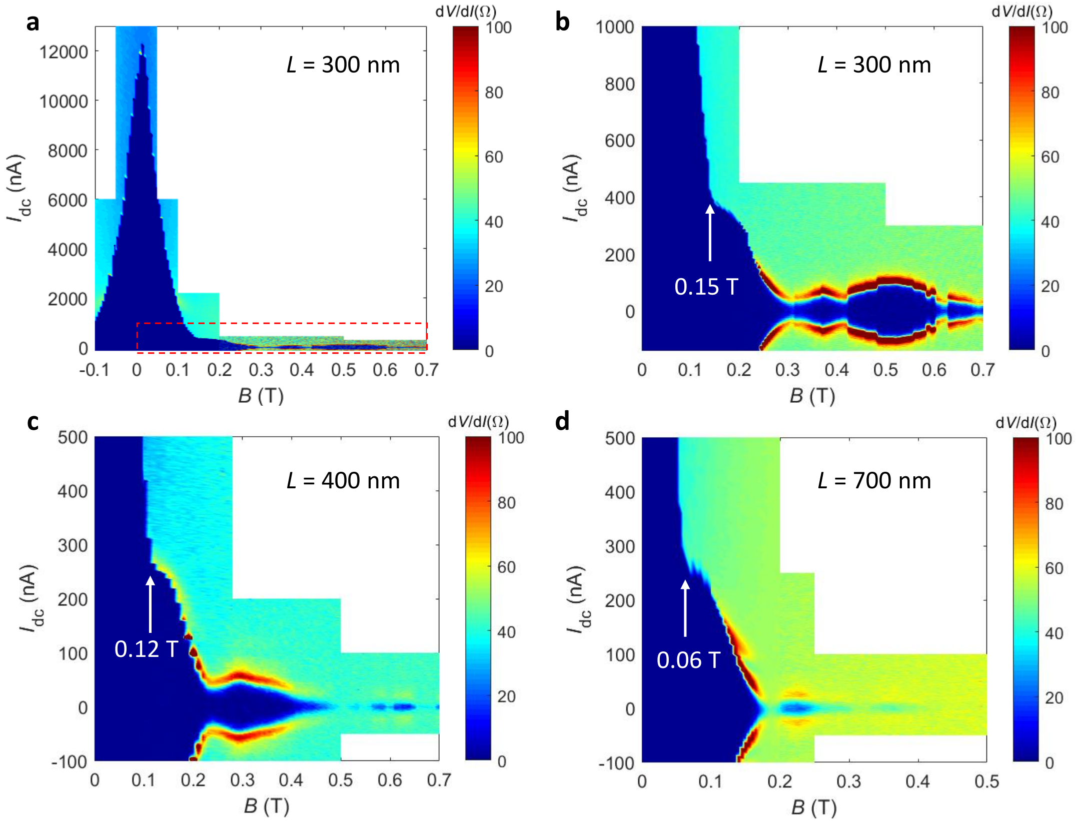

Supplementary Figure 4 | Josephson supercurrent under an in-plane magnetic field perpendicular to the current direction. (**a**) Differential resistance d*V*/d*I* as a function of magnetic field **B** and I_{dc} in a junction with L = 300 nm. The magnetic field is applied in-plane perpendicular to the current, that is, along the x direction. (**b**) The enlarged d*V*/d*I* map of the red dashed box in **a**. Irregular critical current oscillations are observed. The white arrow indicates the location of the first node. (**c-d**) The enlarged d*V*/d*I* map in junction with L = 400 nm and 700 nm, respectively.

For an in-plane magnetic field perpendicular to the current direction, the first node of the oscillation appears at $\mathbf{B}^*$ = 0.15 T for a junction with L = 300 nm. With the increase of channel length L, the location of the first node shifts to lower magnetic field, i.e. $\mathbf{B}^*$ = 0.12 T with L = 400 nm and $\mathbf{B}^*$ = 0.06 T with L = 700 nm. Such a length dependence of critical oscillations is consistent with the Fraunhofer diffraction pattern, and is inversed to that for an in-plane magnetic field along the current direction.

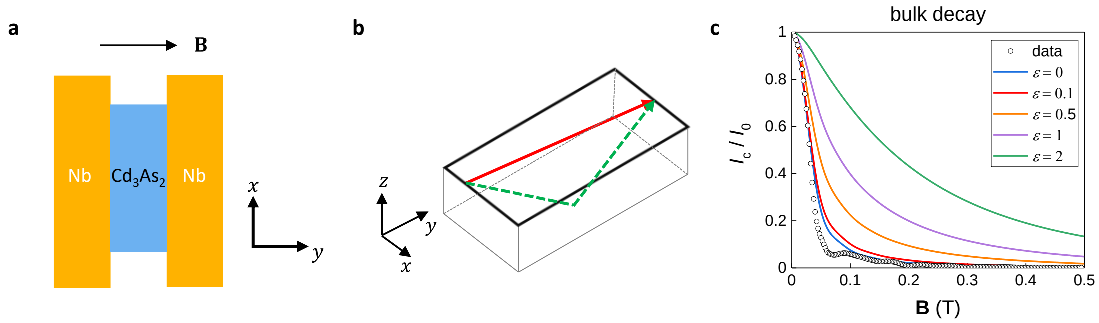

Supplementary Figure 5 | In-plane field orbital effect from bulk states. (**a**) Schematic of the measured Nb-Cd_3As_2 nanoplate-Nb Josephson junction. (**b**) The trajectories of pairing electrons inside the nanoplate sample. Red line indicates the surface trajectory, while green line corresponds to the bulk trajectory with a reflection. (**c**) $I_\mathrm{c}(\mathbf{B})$ oscillations of only bulk state contribution with different decay factors ε and the magnetic field screening factor α = 0.2. The normalized experimental data at V_g = -10 V is plotted in the same scale for comparison. I_0 denotes the critical supercurrent at **B** = 0 T.

Using the model presented in the main text, pairing electrons can acquire the superconducting pairing phase $\phi_1(x_1) - \phi_2(x_2)$ when traversing the junction:

$$\phi_1(x_1) - \phi_2(x_2) = \frac{\pi\alpha\mathbf{B}(x_1 - x_2)t}{\Phi_0}.$$

The magnetic field screening factor α = 0.2 is determined from the fitting of the I_c oscillations by the surface in-plane field orbital effect. We consider the in-plane orbital effect induced I_c modulation with **B**, arising from bulk states:

$$I^{\mathrm{bulk}} = (\Delta\phi, \mathbf{B}) = \int_0^t dt_1 \int_{-\frac{W}{2}}^{\frac{W}{2}} \int_{-\frac{W}{2}}^{\frac{W}{2}} dx_1 dx_2 \frac{1}{\mathbf{r}^\varepsilon} \sin\big(\Delta\phi + \phi_1(x_1) - \phi_2(x_2)\big),\ t \to t_1,$$

where the parameters of the expression I^{bulk} have exactly the same form as those of I^{surface} (refer to the main text), but for bulk state, $\mathbf{r} = \sqrt{L^2 + (x_1 - x_2)^2 + t_1^2}$, $t_1 \in [0, t]$. For bulk pairing states, whatever the decay factor ε is, the in-plane field orbital effects only cause a decay of critical current (Supplementary Fig. 5c).

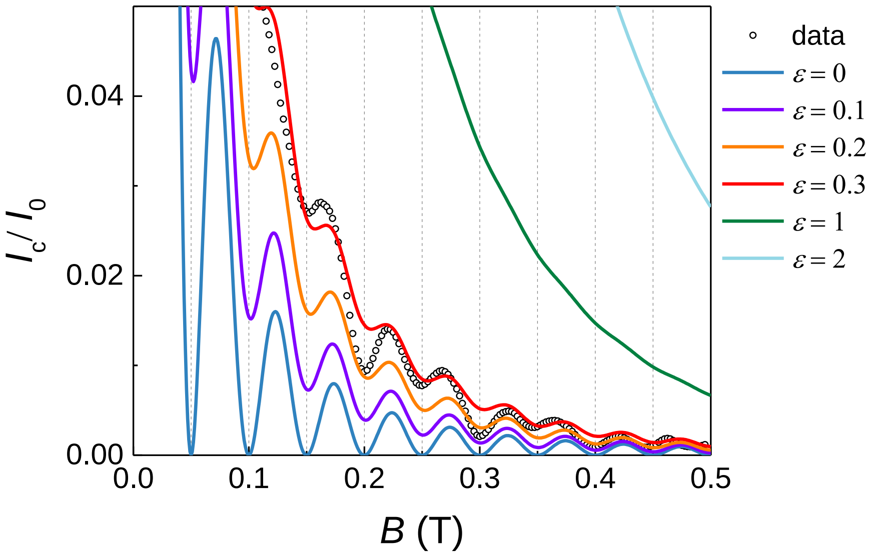

Supplementary Figure 6 | In-plane field orbital effect from surface states with different decay factor ε. The normalized experimental result at V_g = -10 V is plotted in the same scale.

The parameter ε denotes the phase coherent strength along the x direction. The pre-factor $\frac{1}{\mathbf{r}^{\varepsilon}}$ is initially derived from the Green's function of the topological insulator as a description for the length dependence of the order parameter. We extend this into a more generic phenomenological factor, describing the relative contribution ratio of different places to the total supercurrent. When $\varepsilon = 0$, the pre-factor becomes a constant 1, the phase coherence strength is strong along x direction. When $\varepsilon \gg 1$, the pre-factor becomes very small when $\mathbf{r}$ is big, in which the phase coherence strength is weak along x direction.

Supplementary Figure 6 shows the simulations of the $I_c(\mathbf{B})$ with different ε for surface states. When $\varepsilon = 0$ the modulation is 100% that the I_c reaches zero and the amplitude of the damped oscillation is the largest. It decreases when ε increases. The data is approximately best fitted with ε = 0.22 (Fig. 5b in the main text). The small value of ε is consistent with the topological nature of Fermi arc surface states, which could possess relatively long correlation length and thereby strong phase coherence strength.

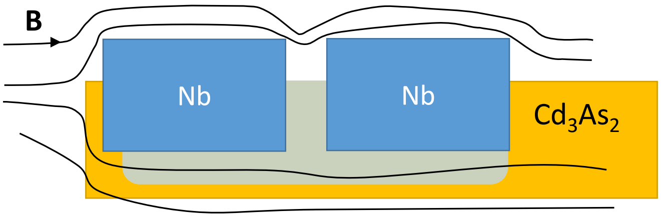

Supplementary Figure 7 | Illustration of the screening effect in devices. The etching process gives an etching depth of Nb electrodes of tens of nanometers.

The parameter α is the screening factor. As a three-dimensional system, the in-plane field have more complicated influence on the devices, since the thickness of the nanoplates cannot be neglected. Based on a rough etching rate of Cd_3As_2, we estimate our samples have etching depth of tens of nanometers. Consequently, the devices experience a relatively large screening effect of the magnetic field and the effective magnetic field is smaller than the applied field (Supplementary Fig. 7). As a result, the devices experienced magnetic field is approximately $\alpha\mathbf{B}$ ($\alpha < 1$) in fact. The value of α can be accessed from the fitting analysis. As the screening effect depends on the device structure, it is not easy to theoretically justify the value of α. Therefore, we simply adopted the value determined from the fitting.

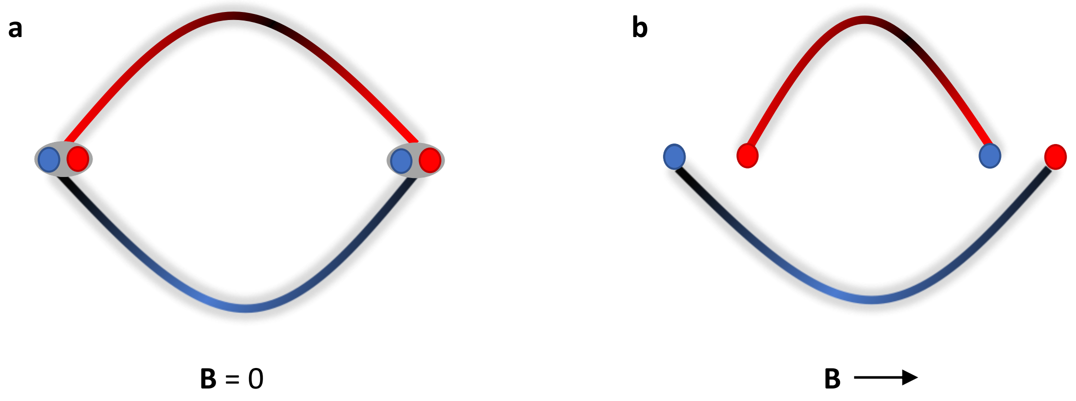

Supplementary Figure 8 | Schematic diagram of Fermi arcs in Cd_3As_2. (a) Without an external magnetic field, Fermi arc connects the surface projections of two Dirac points. Each Dirac point can be viewed as a composite of two degenerate Weyl nodes. (**b**) With an external magnetic field, each Dirac point splits into two Weyl nodes along the direction of magnetic field. Each surface Fermi arc connects two Weyl nodes with opposite chirality. The Fermi arc surface states are no more degenerate. The red and blue balls in **a**, **b**, represent Weyl nodes of opposite chirality.

The two Fermi arcs in Supplementary Fig. 8b are with opposite chirality. Magnetic field results in that the two Fermi arcs are with differnet size. Thus, the chirality balance is broken and there is a polarization of the Fermi arcs.
